# Anaphylactic Reactions to Oligosaccharides in Red Meat: a Syndrome in Evolution

**DOI:** 10.1186/1476-7961-10-5

**Published:** 2012-03-07

**Authors:** Hana Saleh, Scott Embry, Andromeda Nauli, Seif Atyia, Guha Krishnaswamy

**Affiliations:** 1 Department of Internal Medicine, Division of Allergy and Clinical Immunology, Quillen College of Medicine, East Tennessee State University, P.O. Box 70622, Johnson City, Tennessee 37614-0622, USA; 2 Division of Health Sciences, East Tennessee State University, Johnson City, Tennessee, USA

**Keywords:** Galactosyl-(1,3) galactose, Oligosaccharides, Cetuximab, Anaphylaxis, Food allergy, Delayed hypersensitivity

## Abstract

**Objective:**

While most allergic responses to food are directed against protein epitopes and occur within 30 minutes of ingesting the allergen, recent studies suggest that delayed reactions may occur, sometimes mediated by IgE antibodies directed against carbohydrate moieties. The objective of this review is to summarize the clinical features and management of delayed hypersensitivity reactions to mammalian meat mediated by IgE antibodies to galactose-alpha 1,3-galactose (alpha-gal), an oligosaccharide.

**Methods:**

A PubMed search was conducted with MeSH terms: galactosyl-(1,3) galactose, oligosaccharides, cetuximab, allergy/hypersensitivity, and anaphylaxis. Reported cases with alpha-gal-mediated reactions were reviewed. This research study was approved by the Institutional Review Board of East Tennessee State University.

**Results:**

Thirty-two cases of adults presenting with red-meat induced allergy thought to be related to oligosaccharides have been reported in the literature so far, making this a rare and evolving syndrome. Most of these patients demonstrated delayed reactions to beef, as was seen in the case reported by us in this manuscript. IgE specific to alpha-gal was identified in most patients with variable response to skin testing with beef and pork. Inhibition studies in some cases showed that the IgE antibodies to beef were directed towards alpha-gal in the meat rather than the protein. The patients often reported history of tick bites, the significance of which is unclear at present. Reactions to cetuximab, a monoclonal antibody, are mediated by a similar mechanism, with IgE antibodies directed against an alpha-gal moiety incorporated in the drug structure.

**Conclusion:**

Alpha-gal is an oligosaccharide recently incriminated in delayed anaphylactic reactions to mammalian meats such as to beef, pork, and lamb. It appears that anaphylactic reactions to the anti-cancer biological agent, cetuximab, may be linked mechanistically to the same process. More studies are required to understand the underlying molecular basis for these delayed reactions in specific, and their broader implications for host defense in general.

## Introduction

### Beef Allergy and the New Evolving Syndrome

Food allergy remains a well-recognized problem that affects people of different ages and can alter their quality of life [1,2]. Its prevalence and incidence seem to be increasing over the past years [3-7] with more cases of food-induced anaphylaxis being reported [8-11]. Food hypersensitivity reactions are usually mediated by IgE antibodies against the incriminated food allergens such as eggs, seafood, milk, tree nuts, peanuts, wheat, soy, and rarely beef (Additional file 1: Table S1) [8,10-13]. The development of IgE-mediated reaction to a food that was well tolerated in the past sometimes constitutes a true diagnostic and therapeutic challenge to the patient and the physician [14]. The prevalence of food allergy has been increasing, with up to 4% of children having allergic reactions to one or more foods, of which reactions to peanut, soy, wheat, and seafood are probably most common [8,10-15]. While clinical tolerance to food allergens occurs in many children, some such as peanut and shell fish are characterized by the tenacity of the sensitization and persistence of sensitivity into adulthood [12,13,15-17].

Among food allergies, hypersensitivity to red meat is less common [18-22]. It was not adequately studied until recently, with a few reports emphasizing the probable cross reactivity between beef, cow's milk, and other types of red meat [23-31]. Some of the reactions, referred to as "pork-cat syndrome", involve cross-reactivity between cat epithelial allergens and pork [32-39]. The presentations of allergic reactions to meat, as reported in the literature, are reviewed in Additional file 2: Table S2. These include allergic reactions to meat protein, oral allergy syndrome (food-pollen syndrome), the pork-cat syndrome as explained earlier, and some forms of exercise-induced anaphylaxis [18-41].

It was assumed that reactions to mammalian meats would be immediate, and due to IgE directed against specific protein allergens such as bovine serum albumin (BSA) [22,27,42-46]. However, over the past few years investigators have described a new syndrome characterized by delayed reactions to mammalian meat associated with IgE antibodies directed against oligosaccharides [14,47,48]. Chung et al. first reported on IgE antibodies specific to galactose-alpha 1,3-galactose (alpha-gal), an oligosaccharide present in non-primates [49,50], when studying allergic reactions in cancer patients treated with cetuximab [51]. Commins et al. later reported on the role of oligosaccharides and IgE antibodies to alpha-gal in allergy to red meat [14,47,48]. This article will review 31 cases of delayed beef reactions from around the world [18,48,52,53], along with one case from our clinic. It summarizes the current understanding of this rare, novel, and evolving syndrome.

### Historical Perspectives in the Discovery of Alpha-gal-mediated Allergy

**Figure 1:**
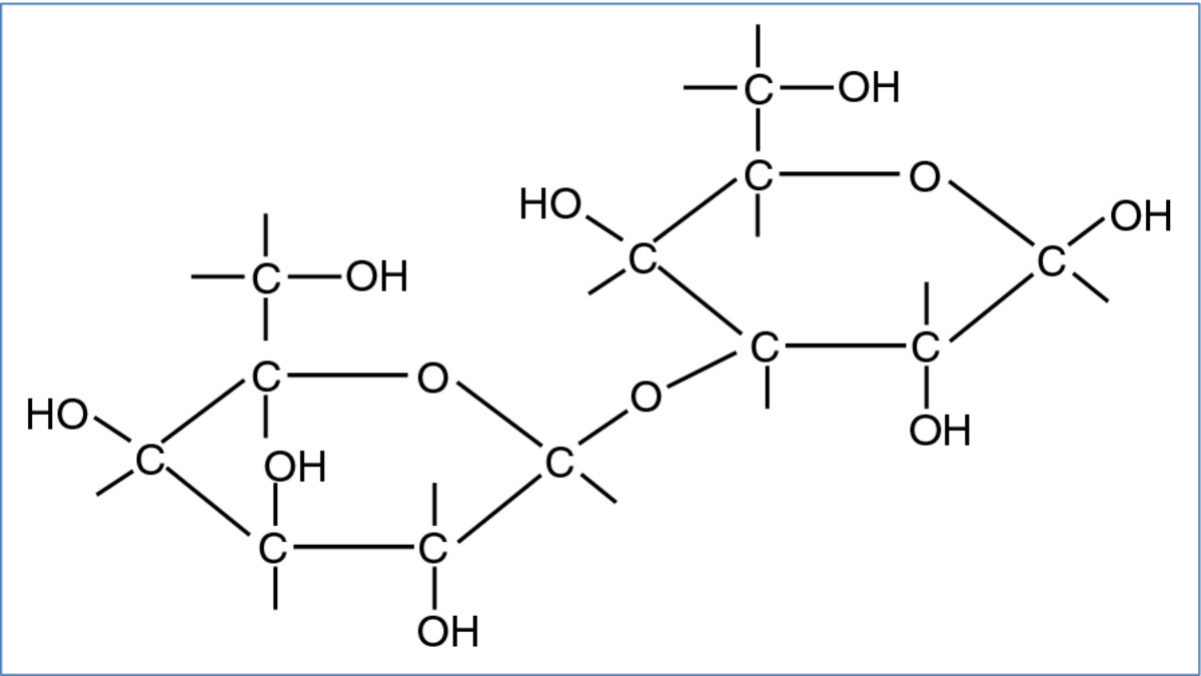
**Structure of galactose-alpha 1,3-galactose (alpha-gal)**. Alpha-gal is an oligosaccharide found in mammalian cells of non-primates [49,50]. The alpha-gal epitope is present in beef, pork, lamb [14,47-49], and cat dander [54,55], and absent in chicken and fish [49]. Beta-galactosyl alpha 1,3 galactosyl transferase, the enzyme needed for formation of alpha-gal, is inactivated in humans and higher mammals [49,56].

Alpha-gal (Figure 1) is an oligosaccharide found in mammalian cells of non-primates [49,50]. The alpha-gal epitope is present in beef, pork, lamb [14,47-49], and cat dander [54,55], but is absent in chicken and fish [49]. Beta-galactosyl alpha 1,3 galactosyl transferase, the enzyme needed for formation of alpha-gal, is inactivated in humans and higher mammals due to an evolutionary process. As a result, immunocompetent individuals may form IgG isotype antibodies to alpha-gal [49,56]. These antibodies contribute to immediate rejection of xenotransplants such as with "pig organs" in humans (recipients). At the same time, the high immunogenicity of alpha-gal may allow for the generation of anti-viral vaccines, as well as tumor vaccines that also carry the alpha-gal epitope [49].

Exposure to food allergens (including perhaps alpha-gal in mammalian meat) results in the generation of IgG antibodies from B cells and hence development of immune tolerance. In predisposed individuals, due to possible genetic/environmental factors (such as a fatty diet, or tick bites), exposure of antigen presenting cells to alpha-gal leads to Th2 activation and induction of interleukins, leading to IgE formation by B cells. This culminates in mast cell activation, eosinophilia, and the full gamut of IgE-mediated hypersensitivity reactions characterized by urticaria, angioedema, and progression to systemic anaphylaxis in some patients [8,57,58].

### Cetuximab; Introduction, Infusion reactions, and Link to Meat Allergy

**Figure 2:**
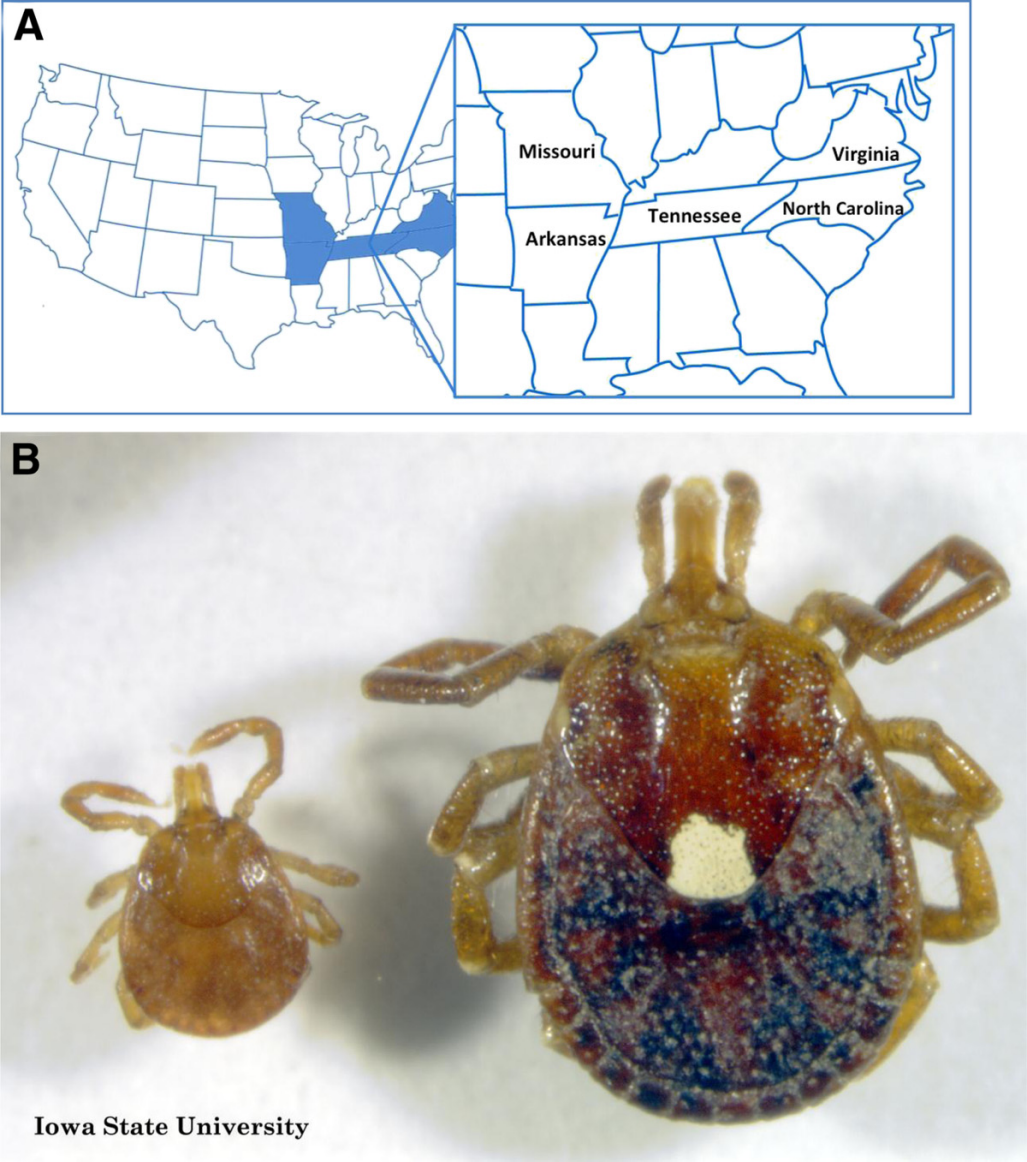
**Areas in USA where IgE to alpha-gal is common and the associated tick**. A: Map of United States of America with highlights on the south-eastern region in which reactions to cetuximab [51], as well as seropositivity to alpha-gal in delayed red meat allergy [48], are most prevalent. **B**: Lone star tick (*Amblyomma *americanum), most prevalent in southeast USA and linked to alpha-gal allergy [74-76]. Image used with permission from Iowa State University Entomology department; Credit to John VanDyk.

**Figure 3:**
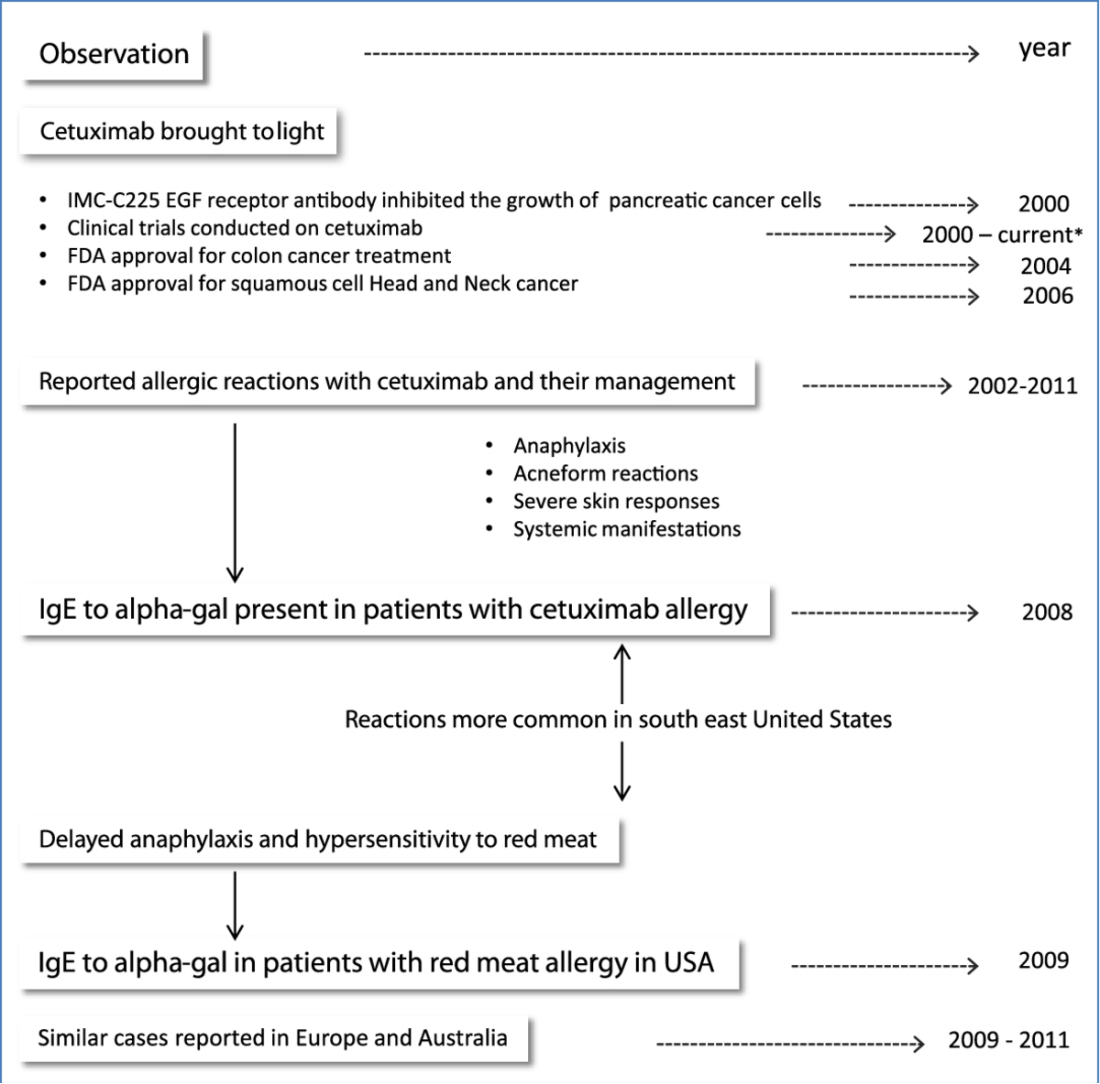
**The events in the understanding of cetuximab and Alpha-gal-mediated hypersensitivity**. With the use of cetuximab in treatment of head and neck cancer and colorectal carcinoma, multiple infusion reactions were reported. Chung *el al*. in 2008 were able to identify IgE to alpha-gal directed to the Fab portion of cetuximab and was related to the allergic reactions. IgE to alpha-gal was later linked to allergic reactions to red meat in America and Europe. Information from references: [18,51,52,59-74].

Cetuximab is a recombinant chimeric epidermal growth factor monoclonal antibody approved for treatment of metastatic colorectal and head and neck cancer [51,59-61]. Initial studies on cetuximab started in 2000, when IMC-C225, was shown to inhibit the growth of pancreatic cancer cells [62]. Further studies and clinical trials on this monoclonal antibody, later given the name cetuximab, were conducted [63-66]. It was shown that cetuximab binds to tumor cells, and is able to activate natural killer cells, eosinophils, and neutrophils to target them against these cancer cells [67]. In 2004, cetuximab was approved for the treatment of colon cancer (http://www.fda.gov/NewsEvents/Newsroom/PressAnnouncements/2004/ucm108244.htm) [59], and subsequently for treatment of squamous cell head and neck cancer in 2006 (http://www.fda.gov/NewsEvents/Newsroom/PressAnnouncements/2006/ucm108609.htm). Since 2002, cetuximab has induced severe hypersensitivity reactions [51,68-70] that have led physicians to further study the nature of these reactions, and determine the appropriate management [71-73]. In 2007 and 2008, reports showed that the infusion reactions were more prevalent in Southeastern United States [51,68] (Figure 2A). This distribution is of interest, and may be related to other factors, including diet as well as exposure to the Lone Star Tick [74] (Figure 2B). In 2008, Chung et al. identified IgE antibodies to alpha-gal in patients who developed mild to severe allergic reactions, including anaphylaxis, following treatment with cetuximab [51]. Then, in 2009, Commins et al. reported on a similar geographic distribution of patients presenting with delayed allergic reaction to red meat, and studies were able to detect IgE to alpha-gal in these patients as well [48]. More patients with this interesting syndrome were further studied in Europe in 2009 and 2011 [18,52]. Figure 3 summarizes the chronological events starting with the discovery of cetuximab, the allergic reactions reported, the role of alpha-gal in these reactions, and the link to red meat allergy.

### The Cetuximab Hypersensitivity Syndrome

Reactions to biological agents (and most drugs) can be classified as IgE and non-IgE mediated reactions, and can present in the form of skin, pulmonary, or cardiac manifestations [77-79]. Non-IgE reactions may be secondary to cytokine release and/or tumor lysis syndrome [80-84]. In the case of cetuximab, evidence suggests that these reactions are IgE mediated and represent true anaphylaxis.

**Figure 4:**
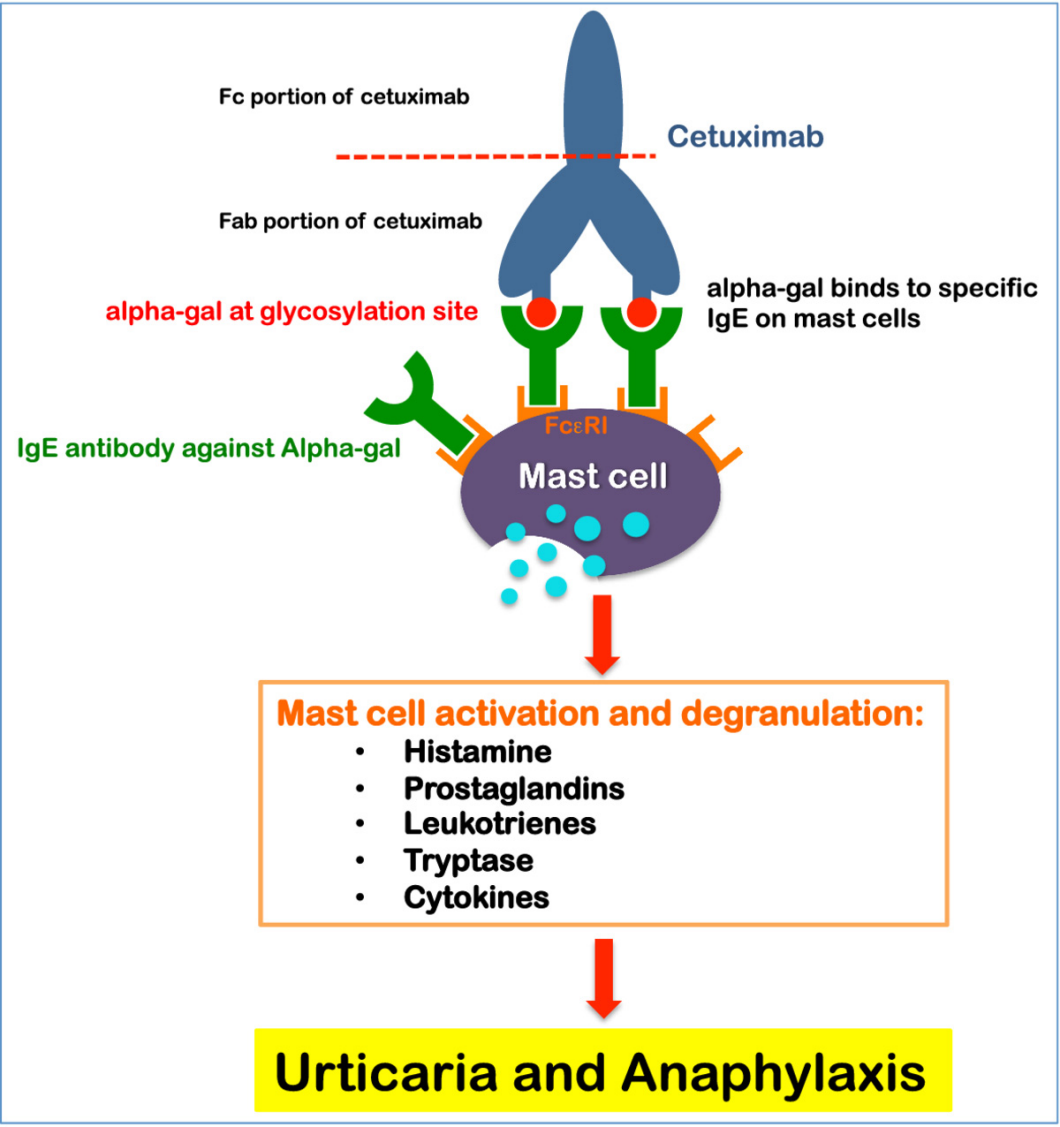
**The possible mechanism behind cetuximab induced allergies**. Cetuximab is a recombinant chimeric epidermal growth factor monoclonal antibody approved for treatment of metastatic colorectal and head and neck cancer [51,59-61]. Infusion reactions with cetuximab are linked to the presence of IgE antibodies directed against the alpha-gal component of the Fab fragment of cetuximab heavy chain [51,85]. Each cetuximab molecule contains two alpha-gal epitopes that can cross-link the high affinity receptor for IgE (FcεRI) on mast cells [51] leading to mast cell activation and release of hypersensitivity mediators [57].

As mentioned earlier, Chung et al. identified IgE antibodies to alpha-gal in patients presenting with severe infusion reactions to cetuximab [51]. In this series, the authors reported on the presence of IgE antibodies against cetuximab in sera from four groups of patients: 76 patients treated with cetuximab in the South-Eastern United States, 72 healthy control subjects in Tennessee, 49 control subjects with cancer in California, 3 treated with cetuximab, and 341 control subjects in Boston. The authors show that 25/76 subjects treated with cetuximab in Tennessee had clinical hypersensitivity to the drug, and samples from 17 of those demonstrated IgE antibodies to cetuximab, compared to only 1/51 in those patients who did not have a hypersensitivity reaction. The IgE antibodies were also found in 15/72 control subjects in Tennessee and only in 3/49 samples in Northern California and 2/341 control samples from Boston. The studies showed that these IgE antibodies are directed against the alpha-gal component of the Fab fragment of cetuximab heavy chain (Figure 4) [51,85]. A recent study by van Bueren et al. [85] reported the presence of alpha-gal epitopes in the Fc portion of several monoclonal antibodies including infliximab, basiliximab, palivizumab, panitumumab, and cetuximab. However, only cetuximab was found to contain alpha-gal in the Fab region of the heavy chain, and interestingly, was the only drug able to bind to IgE specific to alpha-gal (Figure 4) [85]. IgE to alpha-gal failed to bind to the Fc portion of the drugs due to several factors [85]. This might explain the tolerance of some patients to panitumumab after experiencing severe reactions to cetuximab [86-88].

Each cetuximab molecule contains two alpha-gal epitopes that can cross-link the high affinity receptor for IgE (FcεRI) on mast cells [51]. IgE crosslinking of FcεRI leads to activation of mast cells and degranulation with release of hypersensitivity mediators including histamine, prostaglandins, leukotrienes, tryptase, and cytokines [57]. The possible mechanisms behind the cetuximab reactions are demonstrated in cartoon format in Figure 4.

### Clinical Presentation and Diagnosis of Alpha-gal-related Meat Allergy

The clinical presentation of alpha-gal-related red meat allergy is similar to other food allergies, but has many unique characteristics (Additional file 3: Table S3). Upon presentation with suspected food allergy, history taking remains the initial tool for diagnosis [12,18]. Patients with beef allergy related to alpha-gal have symptoms that are common to other food induced hypersensitivity reactions including urticaria, dyspnea, hypotension, angioedema, or even full blown anaphylactic shock [18,48,52,53], but with the caveat that reactions occur hours after the ingestion of an incriminated food. This sometimes makes the diagnosis difficult. A detailed history including type of ingested foods, time to onset of symptoms, the geographic area of residence as well as history of tick bites, can help in achieving the diagnosis. A patient presenting from the South-Eastern United States with allergy to red meat occurring up to 3-7 hours after ingestion, and with a history of tick bites, would be suggestive of alpha-gal allergy.

In general, confirming the diagnosis of a specific food allergy has many components. Although food challenges remain the "gold standard" for definitive diagnosis [12,13], most physicians would start with basic tests including skin prick test (SPT), intradermal testing, or patch testing with the suspected allergen. In some cases where skin tests could not be performed, serum IgE antibody quantification for the suspected allergen/s is safe and easy [12], but there is high number of false positive results, and the IgE level does not necessarily correlate with the severity of the reaction [8,12]. The approach to delayed food reactions however has some caveats. A food challenge may be unnecessary if the history and serological tests are confirmatory, and may in fact be dangerous. Food challenges are best conducted in research environments and their role is yet to be defined in the delayed reactions to mammalian meats.

In the USA, Commins et al. at University of Virginia Health System are the leaders in studying delayed red meat allergy [14,47,48]. They studied 24 patients with IgE to alpha-gal [48]: 4 initially presented with hypersensitivity to beef, 15 identified from a cohort study of 243 patients, and 5 presenting from a clinic in Missouri. All 24 patients had delayed allergic reactions after ingestion of red meat. Patients were between the ages of 18 and 80 years, 14 males and 10 females, and presenting from southeast United States. In addition to IgE to alpha-gal, total IgE levels, and IgE to beef were measured in the studied cases. 22 out of 24 patients had positive (> 0.35 IU/ml) IgE to beef with variable total IgE levels. Skin testing was performed on 18 patients using both commercial and fresh beef extracts, 13 of which gave positive results. 10 patients had intradermal testing to beef and all gave positive results. Although more than 72% of patients had positive SPT to beef and 100% of tested patients had positive intradermal tests to beef, Commins reported that these reactions were not impressive when compared to the IgE to beef, and the severity of reactions they experienced [48]. Studies showed that fresh extracts of beef and pork contain more alpha-gal amounts than commercial extracts, and hence were able to produce better results [18,48]. This is no surprise as multiple reports showed that the allergenicity of beef changes with the method of processing and heat exposure [26,89-92]. The source of meat and the preparation of the extract used in SPT differ between laboratories and even countries. This indeed can attribute to the variability of results in each study. Almost 80% of the studied patients in Commins paper had no further symptoms of hypersensitivity reactions after avoidance of red meat. The others had fewer manifestations. Detailed information of the involved patients and their laboratory results is outlined in Commins and Platts-Mills original paper [48].

Seven other patients studied in Europe presented with delayed allergy to red meat, and had IgE levels positive for alpha-gal and beef [18,52]. In France, investigations on two patients showed mild SPT reactions to raw beef and pork, but significant skin reaction when tested with cetuximab, even with low concentrations [18]. Nunez et al. in Spain also reported on 5 patients with IgE antibodies specific to alpha-gal, all presenting with severe red meat allergy. All five patients tested positive for IgE to beef, pork, lamb, and rabbit. SPT with raw beef and cetuximab were positive in all five as well [52]. Other cases with similar history and presentation to alpha-gal allergy were reported, but to our knowledge testing for alpha-gal was not conducted [14,53]. Clinical presentations of cases with alpha-gal allergy and variable diagnostic tests are summarized in Additional file 4: Table S4.

**Figure 5:**
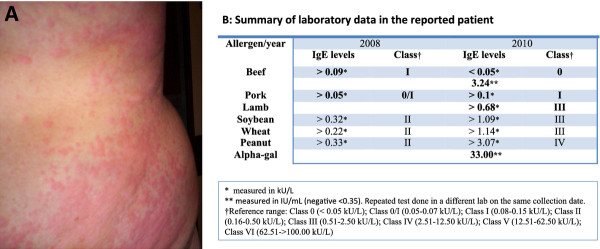
**Urticarial eruptions (A) and laboratory data (B) in a patient with alpha-gal related red meat allergy**. A 48 year-old patient presented with recurrent urticarial eruptions and dyspnea 5 to 7 hours after red meat ingestion. Patient reported history of tick bites 2 weeks prior to his first reaction. Initial workup showed negative IgE to beef and pork. SPT to beef was not performed. Repeated testing was positive to beef and alpha-gal. Patient avoids red meat products and symptoms are well controlled.

A 48-year old male patient with delayed reaction to beef presented to our clinic in East Tennessee. He experienced recurrent urticarial eruptions (Figure 5A) and dyspnea that occurred 5 to 7 hours after red meat ingestion, including beef and pork. Interestingly, the patient reported a history of tick bites 2 weeks prior to his first reaction. Initial blood work showed negative IgE levels to beef and pork, but repeated testing of the same sample was positive to beef. He also had very elevated levels of IgE specific to alpha-gal. Our patient now avoids red meat products and has not experienced any further reactions with food ingestion. Additional laboratory data and information is summarized in Additional file 4: Table S4 and Figure 5B.

### The Presumed Role of Tick Bites

Although genetic factors may predispose an individual to develop an allergic reaction to beef, Commins provided strong evidence that tick bites likely play an important role in this development [48,74]. Bites from the tick species *Amblyomma *americanum (Lone Star tick (Figure 2B)), commonly found in the south east and south central areas of the United States [75], seem to precede the hypersensitivity to alpha-gal[74]. This phenomenon suggests a role in the sensitization process.

The bite of the lone star tick is associated with the development of STARI [76] (southern tick-associated rash illness) usually typified by a large "bulls eye" skin lesion and accompanied by fever, myalgia, and arthralgia. It is important to differentiate this illness from lime disease [76]. How instrumental tick bites are in alpha-gal sensitization, and the mechanism by which this occurs is still unclear.

In a recent study, Commins and James reviewed three patients with known IgE levels to alpha-gal, and documented a significant elevation in these levels after exposure to tick bites [74]. A strong correlation between antibodies to *Amblyomma americanum *and those to alpha-gal was present. Additionally, they screened several individuals and found that most of those whose serum tested positive for alpha-gal, provided a history of tick bites, irrespective of whether they had developed meat reactions [74].

In other areas of the world, history of tick bites (different than *Amblyomma americanum*) is associated with development of meat-induced allergy [52,53]. In a study by Van Nunen in Sydney Australia [53], almost all patients (24/25) presenting with the red meat allergy gave a history of tick bites. The suspected tick is *Ixodes holocyclus *[53], mainly distributed in southeastern Australia [93], and is associated with severe paralysis and cardiovascular problems [94-98]. In Spain 4 of 5 patients with meat allergy expressed history of tick bites prior to the reaction [52]. Although no documented proof of the tick species related to these reactions, the tick common in that area in Spain (north-western) is *Ixodes ricinus *[52]. Cases with reported history of tick bites and the suspected tick are summarized in Additional file 4: Table S4.

### Relationship to Dietary Fat and the Presumed Mechanism of Delay

Another interesting relationship between the areas where alpha-gal allergy is reported in the United States is the obesity rate. The recently reported obesity rates from the Center of Disease Control and Prevention for Tennessee, Arkansas, and North Carolina were higher than those in California and Massachusetts, areas where alpha-gal allergy is uncommon [51]. This raises the question of whether high-fat consumption in diet can be an additional reason for geographical alpha-gal IgE distribution.

The mechanism behind the delayed presentation of symptoms after red meat ingestion in patients with IgE to alpha-gal is still undetermined. A possible explanation suggested by Commins et al. proposes this delay to the time needed for red meat to be digested and presented to circulation [48]. Lipid and glycolipid complexes may mediate delayed absorption and presentation of alpha-gal to antigen presenting cells [14,48]. It only takes about 2 hours for glucose to reach its maximum level in the plasma after a meal. In contrast, it takes about 4-5 hours for dietary triglycerides to reach their peak in circulation [99,100]. In the postprandial state, dietary triglycerides are packaged in the small intestine into very-low density lipoproteins (VLDLs) and chylomicrons, two of the largest lipoprotein particles [101]. Due to their large size, VLDLs (30-80 nm in diameter) and chylomicrons (> 80 nm in diameter) are driven to enter the lymphatic circulation before being emptied into the venous circulation via the subclavian vein. Soluble dietary nutrients, such as amino acids and glucose, directly enter the venous circulation via the superior mesenteric vein/hepatic portal vein. On the other hand, insoluble dietary nutrients including long-chain triglycerides, cholesterol, and lipid-soluble vitamins are packaged by the small intestine into VLDLs and chylomicrons for transport through the lymphatic circulation [102].

Alpha-gal is known to be abundantly present on glycolipids and glycoproteins of non-primate mammals, including the red meat from beef, pork, and lamb [49,50]. Glycolipids and glycoproteins need to be digested in the intestinal lumen before they can be taken up by enterocytes. These alpha-gal-containing digestion products are likely lipid soluble and expected to be transported in VLDLs and/or chylomicrons [48]. The delay of both the allergic reaction and the peak of dietary triglycerides in the circulation, suggests that the allergen from red meat is transported together with dietary triglycerides [48]. Since lipoproteins are made out of a phospholipid monolayer, the alpha-gal containing glycolipids may be inserted into the phospholipid monolayer with the carbohydrate group facing towards the exterior. The "exposed" orientation, rather than "buried", should be optimal for inducing an allergic reaction. Studies should be aimed towards determining the presence of alpha-gal in intestinal lipoproteins. The presence of alpha-gal should only be detected in VLDL/chylomicron fractions from ingestion of red meat from non-primate mammals, and not from other dietary sources, e.g., chicken and vegetarian meals.

### Food Anaphylaxis and Hypersensitivity Reaction Management

The delay in appearance of symptoms related to alpha-gal allergy makes it difficult for patients and physicians to identify the trigger [14]. If such an event were to occur, the patient would be advised to go to the nearest hospital or emergency room. Once the diagnosis is suspected on the basis of history and epidemiological setting, appropriate testing should be done to confirm the diagnosis. Following this, preventive measures include avoidance of red meat and the use of injectable epinephrine [40,48,58,103]. Education and instruction on use of injectable epinephrine is vital. Acute management of patients presenting with anaphylaxis, including those related to alpha-gal, is summarized in Additional file 5: Table S5.

## Conclusion

Alpha-gal allergy is a new and evolving syndrome related to oligosaccharides, rather than protein, in red meat. The IgE mediated response to alpha-gal tends to occur several hours after antigen exposure. This unique presentation constitutes a challenge to both patients and physicians, making detailed history very important if suspected. Reactions to cetuximab seem to be mediated by an identical mechanism. More studies are required about this unique syndrome, including an explanation for the mechanism of delay in presentation and the possible roles obesity and tick bites play in the predisposition for the disorder.

## Abbreviations

Alpha-gal: Galactose-alpha 1,3-galactose; BSA: Bovine serum albumin; SPT: Skin-prick test; FEIA: Fluorescence enzyme immunoassay; VLDLs: Very-low density lipoproteins; FDEIA: Food-dependent exercise-induced anaphylaxis; OSA: Ovine serum albumin.

## Competing Interests

The authors declare that they have no competing interests.

## Authors' Contributions

HS reviewed the literature, generated references, organized the manuscript, and illustrated the figures. SE assisted with manuscript review and corrections. AN assisted in section involving mechanism of delay and role of chylomicrons. SA assisted in section involving mechanism of delay and role of chylomicrons. GK organized the manuscript, edited figures and tables, assisted in discussion, generated references, and participated in the editing and final approval of the manuscript. All authors read and approved the final manuscript.

## Supplementary Material

Additional file 1 **Table 1**. Common food allergens. Click here for file

Additional file 2 **Table 2**. Meat Allergy Types and Features. Click here for file

Additional file 3 **Table 3**. Diagnostic features of alpha-gal-related food allergy. Click here for file

Additional file 4 **Table 4**. Summary of reported cases with alpha-gal allergy in USA and Europe. Click here for file

Additional file 5 **Table 5**. Acute Management and Prevention of Anaphylaxis*. Click here for file
