# Supplementary material for: Anaphylactic Reactions to Oligosaccharides in Red Meat: a Syndrome in Evolution
Source: Clin Mol Allergy. 2012 Mar 7;10:5. doi: 10.1186/1476-7961-10-5 (PMC3402918; doi:10.1186/1476-7961-10-5)
Supplement: Additional file 1 — Table 1. Common food allergens. [file 1476-7961-10-5-S1.DOC]

| **Table 1. Common food allergens** |
| --- |
| Food allergens Peanut  Tree nuts  Cow’s milk  Wheat  Soy  Fish  Crustaceans  Meat* |

*Beef, pork, lamb

Information from references 8,10-13
