# Supplementary material for: Anaphylactic Reactions to Oligosaccharides in Red Meat: a Syndrome in Evolution
Source: Clin Mol Allergy. 2012 Mar 7;10:5. doi: 10.1186/1476-7961-10-5 (PMC3402918; doi:10.1186/1476-7961-10-5)
Supplement: Additional file 2 — Table 2. Meat Allergy Types and Features. [file 1476-7961-10-5-S2.DOC]

**Table 2. Meat Allergy Types and Features**

**Type Clinical Features**

Meat allergy IgE mediated reaction

Immediate symptoms

Positive skin tests or RAST/CAP

IgE to BSA or OSA

Oral Allergy Contact urticarial of mouth following meat ingestion

Pork-Cat syndrome Cross reactivity between cat epithelial allergens and pork leading to reactions

Milk-Beef reactions Cross-reactivity between cow’s milk and beef ingestion

Alpha-Gal sensitivity Reactions mediated by IgE to alpha-gal

Delayed reactions 3-7 hours after ingestion

Prior history of tick bites

FDEIA Exertion-induced anaphylactic reaction to

Ingestion of beef protein

FDEIA: food-dependent exercise-induced anaphylaxis; BSA: bovine serum albumin; OSA: ovine serum albumin; RAST/CAP: in vitro assays for specific serum IgE

Information from references: 18-41
