# Supplementary material for: Anaphylactic Reactions to Oligosaccharides in Red Meat: a Syndrome in Evolution
Source: Clin Mol Allergy. 2012 Mar 7;10:5. doi: 10.1186/1476-7961-10-5 (PMC3402918; doi:10.1186/1476-7961-10-5)
Supplement: Additional file 3 — Table 3. Diagnostic features of alpha-gal-related food allergy. [file 1476-7961-10-5-S3.DOC]

| **Table 3: Diagnostic features of alpha-gal-related food allergy** |
| --- |
| 1. Symptoms suggestive of allergic response*  2. History of red meat ingestion, including: beef, pork, or lamb  3. Delayed onset of the reactions  4. Residential area of presentation** |
| 5. History of tick bites |
| 6. Serological evidence of IgE antibodies to alpha-gal† |
| 7. Skin testing |

* hives, urticaria, angioedema, hypotension, anaphylaxis

** Southeast if in the United States

† galactose-alpha 1,3-galactose
