# Supplementary material for: Anaphylactic Reactions to Oligosaccharides in Red Meat: a Syndrome in Evolution
Source: Clin Mol Allergy. 2012 Mar 7;10:5. doi: 10.1186/1476-7961-10-5 (PMC3402918; doi:10.1186/1476-7961-10-5)
Supplement: Additional file 4 — Table 4. Summary of reported cases with alpha-gal allergy in USA and Europe. [file 1476-7961-10-5-S4.DOC]

**Table 4: Summary of reported cases with alpha-gal allergy in USA and Europe**

| **Number of Cases** | **Age (y)** | **Area or country** | **Type of food** | **Onset of symptoms (in hours)** | **IgE to beef*** | **IgE to alpha-gal*** | **SPT to beef*** | **History of tick bites** | **Tick Species**** | **References** |
| --- | --- | --- | --- | --- | --- | --- | --- | --- | --- | --- |
| **1** | **48** | **TN - USA** | **Beef, pork** | **5 -7** | **+** | **+** | **Not done** | **YES** | ***Amblyomma americanum*** |  |
| **24** | **18-80** | **South-East USA** | **Beef, pork and lamb** | **1-6** | **+ 22/24** | **+ 100%** | **+ in 13/18 tested cases** | **YES∂** | ***Amblyomma americanum*** | **Commins *et al.* [46,83]** |
| **2** | **48** | **France** | **Beef, pork & others†** | **1 and a half** | **+** | **+** | **+** | **Not reported** | | **Jacquenet *et al.* [18]** |
|  | **53** |  | **Pork offal** | **2** | **+** | **+** | **+** |  |  |  |
| **5** | **26-86** | **Northwestern Spain** | **Beef, pork** | **3-6** | **+** | **+** | **+** | **4/5 YES** | ***Ixodes ricinus*** | **Nunez *et al.* [50]** |
| **25** | **17 – 71** | **Sydney -Australia** | **Beef, pork & lamb** | **10/25 after 4 hours** | **IgE to alpha-gal not measured** | | | **YES** | ***Ixodes holocyclus*** | **Van Nunen *et al.* [51]** |

* Values were normalized to (+) or (-). Actual values are available in the references manuscript

** Tick species common in the presenting area

∂ history of tick bites in cases presenting to University of Virginia Health System, not necessary in the 24 reported cases

† lamb, horse & rabbit
