# Supplementary material for: Anaphylactic Reactions to Oligosaccharides in Red Meat: a Syndrome in Evolution
Source: Clin Mol Allergy. 2012 Mar 7;10:5. doi: 10.1186/1476-7961-10-5 (PMC3402918; doi:10.1186/1476-7961-10-5)
Supplement: Additional file 5 — Table 5. Acute Management and Prevention of Anaphylaxis*. [file 1476-7961-10-5-S5.DOC]

| **Table 5: Acute Management and Prevention of Anaphylaxis*** |
| --- |
| Maintain airway and circulation |
| Injectable epinephrine |
| Diphenhydramine |
| Corticosteroids |
| Allergy identification bracelet |
| Avoidance of red meat products including beef, pork and lamb |
| Referral to a specialist for further management |

* in cases where red meat allergy related to alpha-gal is suspected
